# Co-Evolution of Intrinsically Disordered Proteins with Folded Partners Witnessed by Evolutionary Couplings

**DOI:** 10.3390/ijms19113315

**Published:** 2018-10-25

**Authors:** Rita Pancsa, Fruzsina Zsolyomi, Peter Tompa

**Affiliations:** 1 Research Centre for Natural Sciences of the Hungarian Academy of Sciences, Institute of Enzymology, 1117 Budapest, Hungary; 2 Department of Biochemistry and Molecular Biology, Faculty of Medicine, University of Debrecen, 4032 Debrecen, Hungary; zsolyomi.fruzsina@med.unideb.hu; 3 Center for Structural Biology, Flanders Institute for Biotechnology (VIB), Vrije Universiteit Brussel, 1050 Brussels, Belgium

**Keywords:** intrinsically disordered, disordered protein, structural disorder, correlated mutations, co-evolution, evolutionary couplings, residue co-variation, interaction surface, residue contact network

## Abstract

Although improved strategies for the detection and analysis of evolutionary couplings (ECs) between protein residues already enable the prediction of protein structures and interactions, they are mostly restricted to conserved and well-folded proteins. Whereas intrinsically disordered proteins (IDPs) are central to cellular interaction networks, due to the lack of strict structural constraints, they undergo faster evolutionary changes than folded domains. This makes the reliable identification and alignment of IDP homologs difficult, which led to IDPs being omitted in most large-scale residue co-variation analyses. By preforming a dedicated analysis of phylogenetically widespread bacterial IDP–partner interactions, here we demonstrate that partner binding imposes constraints on IDP sequences that manifest in detectable interprotein ECs. These ECs were not detected for interactions mediated by short motifs, rather for those with larger IDP–partner interfaces. Most identified coupled residue pairs reside close (<10 Å) to each other on the interface, with a third of them forming multiple direct atomic contacts. EC-carrying interfaces of IDPs are enriched in negatively charged residues, and the EC residues of both IDPs and partners preferentially reside in helices. Our analysis brings hope that IDP–partner interactions difficult to study could soon be successfully dissected through residue co-variation analysis.

## 1. Introduction

Protein sequences provide rich information on structural and functional constraints in the form of residue co-variation in evolution. With the rapid expansion of available sequence data and computational power, and improvements in global statistical approaches [1], the problem of transitive residue correlations (false positive correlations observed for residues that do not actually contact each other in space) could be largely overcome and the analysis of evolutionary couplings (ECs) between protein residues has achieved important breakthroughs [2,3]. Several groups have demonstrated that the analysis of sequence co-variation can be efficiently used for predicting protein structures [2,3,4,5,6,7,8,9,10], including transmembrane proteins [11,12], defining evolutionary units within proteins [13], and identifying contacting residues of interaction partners [14,15], and interacting subunits of larger complexes [14]. The reason why co-variation analysis does not (yet) provide the ultimate solution to the sequence-based prediction of protein structures is that it requires large, good-quality alignments of sufficiently diverse sequences [16], restricting its applicability to phylogenetically widespread and reasonably conserved proteins.

Intrinsically disordered proteins/regions (IDPs/IDRs) lack well-defined 3D structures, rather, they exist and function as ensembles of rapidly interconverting conformers [17,18,19,20]. The conformational variability and adaptability, extended interaction surface, various embedded interaction motifs [21,22], and post-translational modification sites [23] of IDPs make structural disorder indispensable in regulatory [24], complex-assembly [25], and scaffolding [26,27] functions. IDPs are central to cellular interaction networks [28,29] and are frequently associated with human diseases [30].

IDPs mostly interact with their partners through eukaryotic/short linear motifs (ELMs/SLiMs) comprised of a few specificity-determining residues embedded into a disordered sequence environment that ensures the right positioning of their mostly hydrophobic, crucial interaction residues [21,22,31,32,33]. SLiM-mediated interactions are frequently switched on and off by alternative splicing [31,34,35,36], and are frequently rewired in evolution [31]. As IDPs lack a well-defined tertiary structure and their functional modules are restricted to a few critical residues, most of their sequences are under limited structural and/or functional constraints. As compared to folded domains, this inherent freedom leads to increased rates [37] and altered types [38] of residue changes in evolution, hampering both the identification and correct alignment of homologous IDPs. Despite their indisputable importance, true IDPs (with the exception of ribosomal proteins [15]) and their interactions were not subject to high-scale residue co-variation analyses [6,7,9,11,12,14,15] until very recently. Although disordered regions have less co-varying residues than folded domains [39], Toth-Petroczy and colleagues have recently refined their method, EVfold, to predict potential structured states of IDPs through detecting ECs within their chains [40]. The prevalent phenomenon of induced folding or disorder-to-order transition [41] in IDP-partner recognition, however, suggests that the (partly) structured states of IDPs are encoded not only in their own sequence but also in that of the partner. It follows then that co-evolutionary signals in the interface of the IDP and its partner may actually be just as pronounced as those within a protein fold or between two folded interaction partners.

Therefore, to see if functional constraints originating from partner binding resulted in detectable interprotein ECs of (at least for certain phylogenetically wide-spread) IDPs and to characterize the respective protein pairs, interfaces, and their co-evolved residue pairs, we performed a targeted screen to identify such cases.

## 2. Results

Even though bacterial proteomes are relatively poor in IDPs/IDRs [42,43,44], we restricted our search to bacterial IDP–partner interactions to ensure a sufficient number and diversity of orthologous sequences for residue co-variation analysis. Although there are already a few hundred eukaryotic genomes available, they are not evenly distributed among phylogenetic groups (i.e., a large fraction of them are from mammals) and thus do not show enough sequence diversity on the level of proteins. This is well supported by the fact that recent large-scale residue co-variation analyses have all focused on bacterial protein complexes [9,14,15]. Therefore, we analyzed the 42 bacterial IDPs bound to their folded partners available in the Database of disordered binding sites (DIBS) [45] because there the structural states of the constituent protein chains are backed by experimental evidence. After getting rid of 4 redundant structures and one where the IDP chain was only four residues long, the phylogenetically fairly wide-spread 19 complexes (those with >130 sequences for the IDP in Pfam database 31 [46] full alignments) were selected for co-variation analysis (Supplementary Tables S1 and S2). This way, species–specific complexes, such as virulence factors, certain toxin–antitoxin, effector–chaperone and effector–immunity protein pairs could be counter-selected instead of needlessly occupying co-evolution analysis servers. The sequences were trimmed for the interacting regions/domains or extended to reach the minimum length of 30 residues required for Gremlin analysis as indicated in Table 1, and the Gremlin [15] and EVcomplex [14] servers were used to identify interprotein ECs (See Methods for further details). These methods perform co-variation analyses along similar lines. They first prepare a so-called paired alignment for the protein pair provided, which means that they detect the closest homolog of each of the two provided proteins in each analyzed proteome, and build an alignment wherein the interacting sequences are linked together and filtered for similarity. If the resulting paired alignment has enough sequences, they proceed with detecting co-varying residue pairs (evolutionary couplings, ECs) therein. They use somewhat different approaches to score the pairs of residues based on evolutionary co-variation. From the outputs we exclusively take interprotein ECs, where the coupled residues come from the two different proteins because those provide clues on the interaction. Gremlin could be successfully run on 13 complexes (Table 1), while it stopped in 6 cases due to the alignments being insufficient for analysis. For 7 of the 13 successfully analyzed complexes, Gremlin detected coupled interprotein residue pairs with a scaled score ≥1.3 and probability in the top 12% (*p* > 0.88), hereafter referred to as ECs. For EVcomplex, interprotein ECs with an EVcomplex score >0.9 have been accepted as ECs. EVcomplex also identified ECs for 7/19 complexes (Supplementary Table S1). Both the identified complexes and the detected ECs showed a good overlap between the two methods. Since Gremlin ran the sequence search on a more up-to-date sequence database, it obtained better sequence coverage values and consequently identified more ECs than EVcomplex for almost all the analyzed protein pairs. Based on their residue–residue distances, almost all Gremlin EC pairs fell spatially close, implying that they could be correctly identified co-varying pairs. Thus, we decided to continue the analysis and show the results for Gremlin ECs. The ECs were then checked by PDBe PISA [47] in order to see if they are at the interface (IF) and if they engage in physical interaction (hydrogen bonds or salt bridges; see Table 1).

### 2.1. ECs Were Detected Outside the Sequence Ranges with PDB Coordinates, but Visible ECs Reside on the Interfaces

Gremlin identified 31 ECs in 7 complexes (Supplementary Tables S1 and S3). Intriguingly, for 9 of these co-varying residue pairs, at least one of the constituent residues (mostly the IDP residue) fell outside the segments with PDB coordinates (invisible ECs; marked with N/A in Supplementary Table S3). These invisible ECs make up a remarkably high percentage of all identified ECs. Based on their Gremlin scores and probabilities, they do not seem to be mistakenly predicted pairs as they often have higher scores and probabilities than interface residue pairs with several atomic contacts. Of the 10 invisible EC residues, 5 were present in the respective PDB sequences but had no atomic coordinates, while the other 5 were not included in the constructs used for structure determination. High occurrence of these invisible EC residues suggests that the IDP segments used for structure determination and interaction analyses are often too short, lacking residues that could still form important contacts with the partner. There are actually several instances where the contribution to binding of segments that are disordered in the partner-bound state has been proven, such as binding of CREB to CBP [48] and binding of SF1 to U2Af65 [49], which forms the basis of the concept of fuzzy interactions [50].

**Figure 1:**
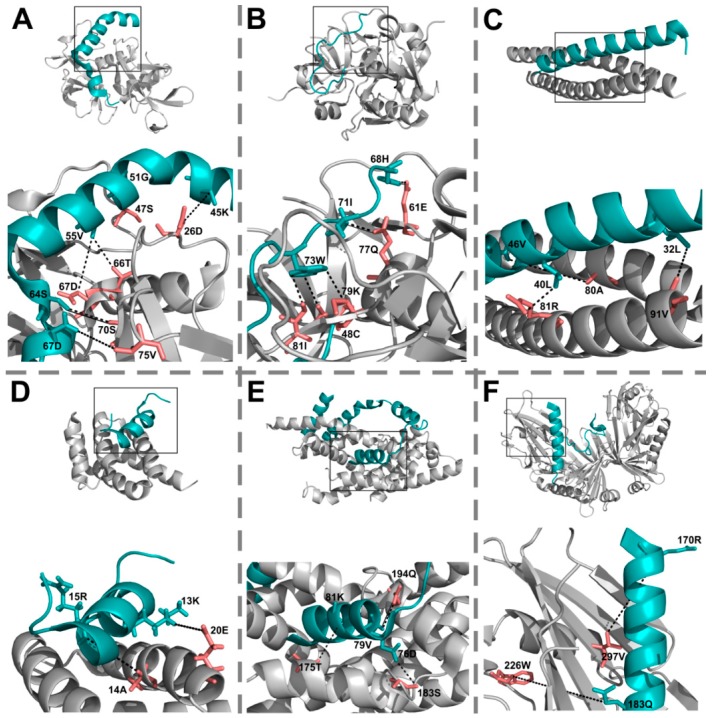
High-scoring interprotein ECs indicating IDP-partner co-evolution. The six protein complexes with multiple high-scoring interprotein ECs predicted by Gremlin are introduced: (**A**) PDB complex 3HPW; (**B**) 5CQX; (**C**) 3M91; (**D**) 2A7U; (**E**) 1SC5; (**F**) 3M4W. IDPs are depicted in teal cartoon style, while the partners are in light grey. Rectangles indicate the part of the complexes which are depicted as detailed interaction maps below. In the interaction maps, the EC side chains are depicted as sticks colored teal in the IDP and salmon in the partner. The interprotein ECs have their respective residue side chains labelled and are connected by dashed lines. For the few pairs forming direct H-bonds or salt bridges according to PISA, the connecting lines are doubled. The 183Q-226W IDP-partner EC pair on the F panel had a distance 17.5Å, thus it was handled as an outlier. None of the depicted complexes represent SLiM-mediated interactions, as we could not identify any ECs for those (see Section 2.5 for further details).

All except one of the 22 visible IDP EC residues (Table 1, Figure 1) are interface residues according to PDBe PISA, and 12 of them are assigned as forming a direct H-bond or salt bridge (or both) with the partner (Table 1), although not necessarily with their corresponding EC pair (Supplementary Table S3). These values already support the validity of the predicted ECs, nevertheless, we decided to investigate them in more detail.

### 2.2. EC Pairs Have More Atomic Contacts between Them than Other Pairs

**Figure 2:**
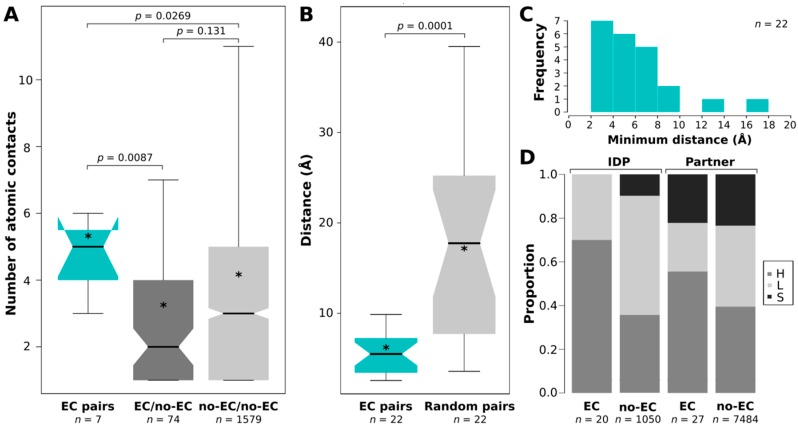
Atomic contacts and distances of ECs. (**A**) The numbers of atomic contacts connecting EC pairs according to PCA compared to those of other contacting residue pairs. Pairwise comparisons were done with Mann–Whitney U test with the corresponding p-values indicated; (**B**) the minimum distances between visible EC pairs are compared to those of randomly selected and paired IDP–partner interface residues; (**C**) histogram showing the distribution of minimum distances between visible ECs; (**D**) the proportions of main secondary structure element types (H—helix, S—strand, L—loop) are compared between EC and no-EC residues for IDPs and partners separately. In the boxplots stars (*) indicate the average values of the datasets.

Interchain residue–residue atomic contacts have been obtained from the Protein Contacts Atlas (PCA) [51] portal for the 34 X-Ray structures. Among EC-containing complexes, 2A7U is an NMR structure so it was not included in the following residue-contact analysis. From the contact lists between the IDP chain and the relevant partner chains, the numbers of atomic contacts between each contacting residue pair have been obtained, the residue pairs have been grouped as ECs, EC residue/no-EC residue pairs and no-EC residue/no-EC residue pairs, and the numbers of atomic contacts for these three groups have been compared. Although only 7 of the 20 visible ECs of X-ray structures had atomic contacts in PCA, with a median of 5, ECs had significantly more contacts between them than contacting residue pairs in the other two groups (Figure 2A). A likely reason behind EC/no-EC pairs having significantly less contacts than no-EC/no-EC pairs is that in the EC/no-EC dataset the contacting residues of EC residues were skimmed. Their respective EC pairs were not taken into account in this dataset; still, they had less space to make contacts with other, no-EC residues. We also wanted to see how many contacts EC residues have, regardless of their partner residue. To this end, we added up the total number of atomic contacts (with all contacting partner residues) for all residues in the contact lists for the IDPs and partners separately. These values were then compared between EC and non-EC residues. We did not find significant differences neither for IDPs nor for their partners (Supplementary Figure S1). Thus, we can claim that although the individual residues of IDP-partner ECs do not have more atomic contacts than other residues, ECs taken as pairs do have more atomic contacts with each other than other residue pairs.

### 2.3. ECs Are Significantly Closer to Each Other than Randomly Paired IDP–Partner Interface Residues

We were also interested in the distance distribution of ECs. For the pairs with PCA atomic contacts, we took the shortest atomic contact as residue–residue distance, while for the rest of the visible ECs (including the ones in the NMR structure 2A7U) we calculated the minimal residue–residue distances using PyMOL (Supplementary Table S3). We compared the distance distribution so obtained to an equivalent reference distance distribution of randomly selected and subsequently paired IDP and partner residues picked from the same interfaces and found that ECs are highly significantly closer in space than the randomly paired interchain interface residue pairs (Figure 2B). The sampling of randomly paired interface residues was carried out 100 times and their distances were consistently significantly larger than those of ECs (*p* < 0.01). The descriptive distribution features (minimum, 1st quantile, mean, median, 3rd quantile, maximum) of the 100 samples have shown an average standard deviation of 2.31 which we interpret as low variation among the random samples. 18/22 visible ECs were closer than 8 Å, two were between 8 and 10 Å far, and only two had >10 Å distances (Figure 2C). The latter two were assigned as outliers by R, they probably represent mistakenly identified pairs (or have larger distances due to other reasons [52]) and thus have been handled as outliers and excluded from distance comparisons (Figure 2B). One of the outliers is the 183Q-226W IDP-partner EC pair in the RseA/RseB (PDB: 3M4W) complex as indicated in Figure 1. The other outlier is the sole EC pair (74D-133Y) identified in the CP12/Glyceraldehyde-3-phosphate dehydrogenase (PDB: 3B1K) complex. Therefore, the latter complex is left without any reliable ECs, and thus it is not shown in Figure 1.

### 2.4. EC Residues Preferentially Occur in Helices in Both IDPs and Partners

To assess the structural preferences of EC residues, we have computed DSSP secondary structure assignments for the complexes. The EC residues preferentially resided in helices for both IDPs (*p* = 0.003) and partners (*p* = 0.039) compared to other residues (Figure 2D). Also, the detected interprotein ECs typically cluster in one alpha helix of the IDPs, although in most cases the studied chains did not have more: in anti-sigma-28 factor FlgM they cluster in helix4 (Figure 1E), while in RseA in the longer helix of the two (Figure 1F). Furthermore, ECs have also been detected in the toxin-antitoxin MazF-MazE pair, where the IDP MazE does not fold into a regular secondary structure on the surface of the MazF dimer (Figure 1B).

### 2.5. General Trends Observed for the Protein Complexes with High-Scoring Interprotein ECs

The seven protein pairs yielding high-scoring ECs mostly had >2 Gremlin sequence/residue alignment coverage values, while the ones that stopped running or did not provide high-scoring ECs tended to have values <1 (Table 1). The coverage values did not correlate with the number of IDP homologs in PFAM. Longer-interacting IDP regions with wider phylogenetic spread (for the corresponding phylogenetic groups see Supplementary Table S1) had a higher chance for sufficient coverage values, while short IDP chains that required significant extensions for reaching the minimum length of 30 residues did not have a good chance for >1 coverage values even if mediating a phylogenetically widely conserved interaction. Therefore, the complexes where the IDPs interact with their partners in the typical IDP manner—through ELMs/SLiMs or molecular recognition features (MoRFs)—namely the enolase- and PNPase-interacting motifs of RNase E [53], the SspB-interacting region of the N-terminal part of RseA, and the C-terminal interaction motif of single-stranded DNA-binding protein (SSB) recruiting diverse partner proteins [54,55], did not have enough PFAM sequences for analysis or, despite a vast amount of sequences in PFAM, did not show any high-scoring ECs. The lack of a sufficient number or diversity of detectable homologs for these important interaction motifs/regions could be due to their short length, the relatively small fraction of the actual specificity-determining residues and the fast evolutionary turnover of the surrounding other residues [31].

In the case of the interactions mediated by the SSB C-terminal motif, that are among the phylogenetically most widespread ones, the lack of detected ECs could be attributed to different reasons. The C-terminal motif is 9 residues in length, so it had to be extended by 21 residues from the poorly conserved SSB linker region to reach a total length of 30 residues. The lack of conservation in the linker segment dilutes the information in the motif, whereas the multitude of interaction partners simultaneously restricting the evolution of the motif [55,56] leads to a complete lack of sequence variation in most of its residues, which could both contribute to the lack of detected ECs.

While EC residues showed a strong preference for interface helices, only 58.8% of ELM instances form secondary structure elements, with only 16.2% of them being mostly helical and 7.6% being partially helical according to a large-scale analysis of the eukaryotic linear motif (ELM) database [31]. Although our dataset only contains a few bacterial short linear motifs, which are not part of the ELM database, they show a similar distribution among secondary structure types as proposed for their eukaryotic counterparts. The SSB C-terminal motif does not form a secondary structure with any of its 5 partners, while the SspB binding motif of RseA forms two very short helices. The PNPase- and enolase-binding motifs of RNase E were not subjected to EC analysis because they did not have the sufficient amount of PFAM sequences, but the former binds through beta sheet augmentation, while the latter forms a short helix. Thus, the identified SLiMs do not show a preference for helical conformation, and by mostly spanning only 4–9 residues, even the helix-forming ones are not long enough to form extended helical structures on the surface of binding partners, which could also contribute to the lack of detected ECs. In all, the complete lack of ECs for SLiM-mediated interactions regardless of their phylogenetic spread definitely represents a major limitation of applying residue co-variation-based approaches for the analysis of IDPs.

Although multisubunit enzymes often contain predicted disordered chains that occupy a completely extended conformation in the complex, and such cases would be perfect candidates for IDP-partner co-evolution analysis, the structural features of these subunits are rarely analyzed on their own. Due to this reason, our DIBS-derived dataset only has 4/37 permanent protein complexes, while most of them depict transient interactions (Supplementary Table S1). The seven complexes with identified ECs contained both permanent (1) and transient (6) complexes in similar fractions as seen for the overall dataset, so permanence of the interactions does not seem to largely affect their co-evolution patterns at first glance. However, it is interesting to note that all the complexes with identified ECs had IDP–partner interface areas >1000 Å^2^, except for the sole complex with the permanent interaction of two subunits of the ATP synthase that only had 610.5 Å^2^. This might imply that, in permanent protein complexes, which are phylogenetically widely distributed, co-evolution of interacting subunits is so prominent that it can be detected even if the corresponding interaction surfaces are relatively small.

### 2.6. ECs Could Not Be Identified for Very Small Interfaces with no H-Bonds or Salt Bridges

**Figure 3:**
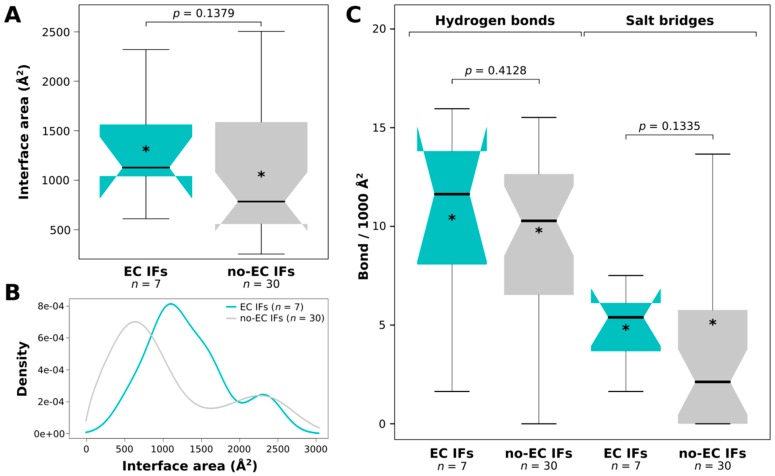
Comparison of interface areas and bonds for EC and no-EC interfaces. (**A**) Comparison of EC and non-EC interface areas with Mann–Whitney U test. (**B**) Density plot of the interfaces in the two groups. (**C**) Comparison of the normalized numbers of H-bonds and salt bridges between EC and non-EC interfaces with Mann–Whitney U test, with the corresponding *p*-values indicated. In the boxplots, stars (*) indicate the average values of the datasets.

By comparing the interfaces of the complexes with and without ECs (the latter group comprised all the 30 non-redundant complexes that did not provide high-scoring ECs by Gremlin) for their interface areas, H-bond and salt bridge densities, we did not find any significant difference (Figure 3). However, it became evident that ECs could not be detected for interfaces <600 Å^2^ (for transient complexes this threshold rather seems as 1000 Å^2^) in size, not even for exceptionally widely conserved interactions. ECs could also not be identified for interfaces without any assigned H-bonds or salt bridges (Figure 3C).

### 2.7. EC Interfaces Are Enriched in Negatively Charged Residues

**Figure 4:**
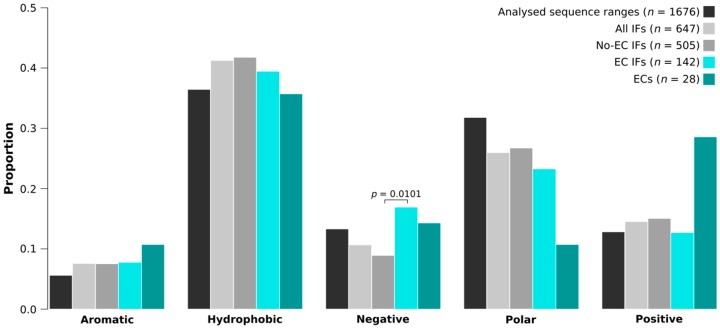
The amino acid group compositions of IDP EC-carrying interfaces and ECs. The amino acid group compositions of trimmed protein segments (analyzed sequence ranges), all interfaces (all IFs), interfaces with no ECs (no-EC IFs), EC-carrying interfaces (EC IFs) and EC residues of IDPs. Both EC-carrying interfaces and EC residues have been compared to all IFs. Also, EC interfaces were compared to no-EC interfaces. P-values are only indicated for amino acid group proportion differences that were found significant by the built-in test of equal proportions of R.

We have also calculated the amino acid and amino acid group compositions of the trimmed protein segments (analyzed sequence ranges), all interfaces (all IFs), interfaces without any detectable EC pairs (No-EC IFs), EC-carrying interfaces (EC IFs) and EC residues for the IDPs and partners separately. Comparing EC IFs and ECs to all IFs, we found no significant differences either for IDPs (Figure 4, Supplementary Figure S2) or their partners (Supplementary Figure S3), although the enrichment of the EC-carrying interfaces of IDPs in negative residues was nearly significant (*p* = 0.0519). We have to note though that we had only 28 IDP- and 30 partner EC residues, which is suboptimal for rigorous statistical analysis. Due to the small sample size, even seemingly very large differences were not statistically significant, for instance, IDP EC residues were not found to be enriched in positive amino acids compared to all IDP interface residues, despite having almost two times as high fraction of positives (28.6% vs. 14.5%; Figure 4). When comparing the compositions of EC interfaces to those of no-EC interfaces, we found that the EC-carrying interfaces of IDPs harbor significantly more negatively charged residues (*p* = 0.0101), in particular glutamates (*p* = 0.0240), than the IDP interfaces with no ECs (Figure 4, Supplementary Figure S2). For the partner interfaces no significant differences were found (Supplementary Figure S3).

## 3. Discussion

Due to increased substitution rates [37], IDP homologs are difficult to identify and correctly align. Also, their extended, floppy conformational states manifest in less contacting residue pairs per unit length compared to folded proteins/domains, also leaving less room for residue co-evolution [39]. Furthermore, IDPs excel in moonlighting [57], meaning that they can interact with multiple partners (often through the very same sequence region) and thus their evolution might well be simultaneously constrained by many different partners. However, these multiple different interactions are only poorly represented in structural databases, and residue co-variation analysis can also only be performed for one pair of sequences at a time. Another important factor is that the ensembles and interactions of IDRs are frequently modulated by post-translational modifications (although these are largely limited to eukaryotic proteins), which currently cannot be taken into account by the available residue co-variation analysis methods. Therefore, we need to acknowledge that, at the moment, it is not feasible to comprehensively cover the full range of structural and interaction plasticity of disordered proteins by such analyses. Due to these reasons, IDPs and their interactions have hardly been investigated for the occurrence of residue co-variation [6,7,9,11,12,14,15]. Although a recent analysis suggested structured states of IDPs based on observable intrachain ECs [40], the co-evolution of IDPs with folded partners remained to be elucidated. To possibly demonstrate and understand IDP–partner co-evolution, we performed a comprehensive analysis of IDP–partner interprotein evolutionary couplings on the available bacterial complexes of the DIBS database using dedicated methods [14,15]. To ensure sufficient diversity and depth of sequence coverage by homologs, we restricted our analysis to bacterial IDPs and their cognate partners. Furthermore, to improve internal consistency of the predictions, we applied two different algorithms, Gremlin and EVcomplex. We could identify high-scoring interprotein ECs for seven interacting protein pairs, typically the ones with the best alignment coverage. Alignment coverage is, however, not strictly correlated with phylogenetic spread; it also depends on the length of interacting protein regions. For complexes where the IDP contacts the partner over a relatively long sequence bit, like, for instance, for the FlgM-FliA complex (Figure 1E), we had a good chance to identify some ECs. However, although SLiM-mediated interactions are highly preferred by IDPs and some of them are exceptionally widely conserved, they did not show high-scoring interprotein ECs, probably due to their short length, wherein the evolutionary information on a relatively small fraction of specificity-determining residues is diluted by many more surrounding other residues undergoing fast evolutionary turnover [31]. The absence of detected ECs for SLiM-mediated interactions could be a major limitation of performing residue co-variation analyses on IDPs, as SLiMs are crucial and widely applied interaction units of IDPs that mediate contacts with many of their partners.

Supporting the validity of the predicted ECs, i.e., that they represent true physical contacts between the IDP and its partner, or at least could be spatially restrained by each other, the majority of the visible ECs fall within 8 Å. Residue contact networks from PCA also showed that several EC pairs had multiple atomic contacts; moreover, ECs had more atomic contacts than other contacting IDP–partner residue pairs. Regarding their structural preferences, EC residues tend to be located in helices in both IDPs and folded partners. Although EC-carrying interfaces did not significantly differ in size or in the surface-normalized number of H-bonds and salt bridges, it is important to note that no ECs could be identified in interfaces that are small (<600 Å^2^) or have no hydrogen bonds or salt bridges, no matter how widely conserved the interaction is. Since for the partners we did not find enrichment in any residue type, we do not claim that the enrichment of EC-carrying IDP interfaces in negative residues would imply the employment of increased electrostatic attraction compared to other IDP–partner interfaces. Therefore, we cannot ascertain the biological relevance of the noted preference for negatively charged residues.

Importantly, ECs could be identified for both permanent and transient IDP–partner interactions. This shows that not only obligatory, permanent associations, such as the subunits of the ribosome [15] or heteromeric enzymes, but also well-established signaling relationships, such as regulatory antisigma factors, have a trace of detectable co-evolution between the IDP and its interaction partner. Regarding the evolutionary history and genomic proximity of the genes encoding the protein pairs with high-scoring ECs, the CcdA-CcdB (PDB: 3HPW) and MazE-MazF (5CQX) toxin–antitoxin pairs, the alpha and delta subunits of the ATP synthase (2A7U) and the Sigma-E/Anti-Sigma-E regulatory factors RseB and RseA (3M4W), are each encoded on common operons. This suggests that these protein pairs are co-expressed and their co-occurrence is strongly preferred in evolution. To the contrary, the other two pairs, FliA with its anti-sigma factor FlgM (1SC5) and proteasome-associated ATPase (product of *mpa* gene) with prokaryotic ubiquitin-like protein Pup (3M91) are located in different operons and are thus substantially less strictly associated both in gene-regulatory and evolutionary terms. Therefore, based on our findings, a phylogenetically widely preserved protein–protein interaction that buries an interface larger than SLiM-mediated interactions typically do, might be enough for the reliable identification of interprotein evolutionary couplings. No other assumption regarding permanence of the interaction or co-regulation of the corresponding genes needs to be made prior to analysis, although such factors could increase the chance of finding true ECs.

In this first dedicated study of IDP–partner co-evolution, we also show that IDPs are difficult to investigate by methods addressing residue co-variation, due to their fast evolutionary changes, limited sequence representation, increased propensity for moonlighting functions, frequent use of short linear motifs for partner binding and relatively few co-evolving residue pairs both within their chains [39,40] and with their partners. Nevertheless, by demonstrating detectable footprints of IDP–partner co-evolution for interactions with largely different functional readouts, our results are also promising. They imply that the explosion in the number of sequenced genomes, the continuous improvement of techniques of sequence homology detection [58], and advances in sequence alignment approaches optimized for IDPs [40] could soon empower residue covariation analyses of IDPs to provide predictions and new insights into the structures and interactions of IDPs whose experimental investigation proved to be challenging.

## 4. Materials and Methods

### 4.1. Dataset Preparation

Bacterial protein complexes were obtained from the DIBS database [45] (Supplementary Table S1). We identified 4 of the 42 as redundant: DIBS: DI2210001–PDB: 3TCJ, DI1200012–3UF7, DI1210005–3C94, and DI1210010–5CW7 were excluded due to redundancy to DI2200001–3HPW, DI1200011–3UF7, DI1210004–3C94, and DI1210007–5CZF, respectively. The DI1200014–5F56 complex was also excluded due to the IDP peptide being <5 residues long. For the remaining 37 complexes, the constituent chains were trimmed or extended to make them optimal for co-evolution analysis. IDP chains shorter than 30 residues were extended based on UniProt [59] to reach the minimum of 30 residues length required for Gremlin analysis. Terminal segments could obviously only be extended to one direction. For the two short IDP chains representing internal segments, more extension was added to the end with residues forming an interface with the partner chain. Partner chains/very long IDP chains were trimmed to interacting domains/subdomains to ensure that the best possible coverage values are obtained (sequence coverage values are highly dependent on the total length of the analyzed sequences). The resulting trimmed UniProt regions used for further analysis are indicated in Table 1. Then for each complex, the IDP counterpart was checked in PFAM 31 [46] to get an idea of their phylogenetic spread. If, for the IDP region in the complex or at least for a neighboring protein domain/region of the protein there were no PFAM families available, or the number of sequences in the full alignments of the relevant PFAM families were <130, then the complex was excluded from correlated mutation analysis (for PFAM families see Supplementary Table S2).

Information on the complexes representing permanent or transient interactions was taken from the literature and UniProt subunit structure annotations. To assign if the genes are encoded within a single operon/gene neighborhood, we used information in the STRING database [60] and Ensembl Bacteria [61]. Assignments on the phylogenetic spread of the interactions were based on literature mining.

### 4.2. Co-Evolution Analysis

The trimmed regions of the 19 remaining complexes were run with the Gremlin [15] and EVcomplex [14] webservers for interprotein co-variation analysis. When using Gremlin, for regions >60 residues the e-value threshold was set to E-06 and the number of iterations with Jackhmmer to 4, while for regions ≤60 residues, we applied a less stringent e-value threshold of E-04 with 8 iterations. The reason for using a less stringent e-value threshold for shorter sequences is explained and supported by the Introduction/Updates section of the Gremlin webserver. Δgene was set to 1–∞ to identify the closest homologs in the analyzed proteomes regardless of their genomic location. In 6 cases, Gremlin stopped due to insufficient alignments for further analysis. It finished the analysis in 13 cases. We accepted interprotein residue pairs with a scaled score ≥1.30 and a probability >0.88 as co-varying pairs; evolutionary couplings (ECs). These thresholds were selected based on our observation that among the predicted possible EC pairs with a scaled score >1.3 the probability values are the main determinants of the residue pairs representing true positive predictions with residue–residue distances reflecting direct contacts or false positive predictions with large interresidue distances. Therefore, we have selected a strict probability value threshold (top 12%) to avoid false positive ECs compromising the dataset used for analysis. The 7 complexes with such high-scoring interface ECs identified by Gremlin were analyzed further and compared to the remaining 30 complexes with no high-scoring ECs.

For the EVcomplex analysis, we applied the default e-value (10^−5^) in searching for homologues and chose the option of selecting the closest homologs of the query sequences from the analyzed proteomes. Here, interprotein ECs with an EVcomplex score >0.9 have been accepted as ECs. EVcomplex identified ECs for 7/19 complexes.

### 4.3. Interface Properties

For each complex, information on the smallest meaningful biological assembly (chains that can represent the biologically relevant interaction) was taken from DIBS and is indicated in Supplementary Table S1. These contain strictly one IDP chain, which interacts with one or more (but in this case identical) partner chains. An exception is 3O0E, where from the DIBS-indicated L and A chains, L was not present in PDBe PISA, so the equivalent M and B chains have been used. Interface areas, interfacing residues, and interchain physical interactions were derived from PDBe PISA [47] assignments. Total interface areas were defined as the sum of interchain interface areas between the IDP chain and the different interfacing partner chains. The total number of IDP–partner interface H-bonds and salt bridges were also obtained as the sum of such bonds between the IDP chain and the interfacing partner chains, then these were normalized for 1000 Å^2^ interface area before statistical comparisons. Interface residues were assigned for each chain based on PISA.

### 4.4. Amino Acid Compositions

In the residue and residue group composition analyses, we have calculated the compositions of the trimmed protein segments (analyzed sequence ranges), all interfaces (all IFs), interfaces with no ECs (no-EC IFs), EC-carrying interfaces (EC IFs) and EC residues for the IDPs and partners separately. Identical partner chains have been only included once into these composition analyses. The interfaces have been defined based on PDBe PISA, so regions of the trimmed protein segments falling outside the segments with PDB coordinates are not represented by the interface composition values. However, they are represented in the analyzed sequence ranges and among ECs. Residue groups were defined as: hydrophobic (G, A, V, L, M, I), aromatic (F, Y, W), polar (S, T, C, P, N, Q), negative (D, E), and positive (K, R, H). Residue group and residue proportion significances were obtained by the built-in test of equal proportions of R [62].

### 4.5. Residue–Residue Contact Networks

Interchain residue–residue atomic contacts have been downloaded from the Protein Contacts Atlas [51] for all the X-ray structures (34/37 complexes) using the default 0.5 Å distance cut-off value in PCA. Only the contact lists between the IDP chain and the relevant partner chains have been used for analysis. The number of atomic contacts between each contacting residue pair has been obtained. Also, the total number of atomic contacts (with all contacting residues) has been calculated for all the residues with at least one such interchain atomic contact. If the same partner residue had atomic contacts with the IDP from more than one partner chain, then those different contact numbers were added up to get the total contact number. Also, for IDP residues, all atomic contacts with partner residues were added up regardless of the partner chain. The calculated totals have been compared between EC and non-EC residues for the IDPs and partners separately.

### 4.6. Secondary Structure Assignments

DSSP secondary structure assignments [63] have been obtained for all the residues present in the complex structures in a way that only one of the identical partner chains has been used. The eight DSSP secondary structure element type assignments have been simplified and grouped in the traditional way into three larger classes: helix (G, H, and I), strand (E and B) and loop (S, T, and C). The distribution of residues among different secondary structure element types have been compared between EC residues and non-EC residues for the IDPs and partners separately using the built-in test of equal proportions of R.

### 4.7. Residue–Residue Distances

For the ECs being in direct atomic contact according to the Protein Contacts Atlas, the shortest atomic contact distance (between heavy atoms) has been accepted as residue–residue distance. For the rest of the ECs, the shortest distance between the heavy atoms of the two residues has been measured using PyMOL (https://pymol.org/2/; The PyMOL Molecular Graphics System, Version 2.0 by Schrödinger, LLC, New York, USA). Then, for each EC we have randomly selected an interface IDP residue and an interface partner residue from the same interface, and measured their distance similarly. The distances of ECs and the thereby assembled equivalent random reference interface pairs have been compared by Mann–Whitney U test. To check if the observed difference remains consistent, we have obtained 100 additional samples of random residue pairs and compared their distances to those of ECs.

## Figures and Tables

**Table 1:** Residue co-variation analysis of IDP–partner interactions with broad phylogenetic spread.

Complex		Folded Partner		IDP				Gremlin Analysis Results	
DIBS ID-PDB ID (Chains Partner(s)|IDP)	IF Area (Å^2^)	Gene Name	Uniprot AC_region	Gene Name	Uniprot AC_Region	Length	# of Seq. in PFAM 31 Full	Coverage (seq/res)	ECs by Gremlin/by EVComplex/Gremlin IDP ECs on IF/in Bonds ^a^
DI4200001-3B1K (BG|D)	1016	*gap2*	Q9R6W2_78-215	*cp12*	Q6BBK3_46-75	30	507	0.76	1/0/1/1
DI2200001-3HPW (AB|C)	1483	*ccdB*	P62554_1-101	*ccdA*	P62552_37-72	36	395	4.06	7/0/6(1 inv ^b^)/2
DI2200002-5CQX (AB|C)	1128	*mazF*	P0AE70_1-111	*mazE*	P0AE72_53-82	30	4349	3.00	9/1/5(4 inv)/5
DI2200004-3M91 (AC|B)	1065	*mpa*	P9WQN5_46-96	*pup*	P9WHN5_21-64	44	511	2.19	5/0/2(2 inv)/1
DI2200006-3M4W (AC|E)	2321	*rseB*	P0AFX9_220-318	*rseA*	P0AFX7_125-195	71	253	1.67	2/0/2/1
DI1200004-1SC5 (A|B)	1641	*fliA*	O67268_1-236	*flgM*	O66683_1-88	88	1450	3.43	3/10/3/1
DI1210003-2A7U (B|A)	611	*atpH*	P0ABA5_1-134	*atpA*	P0ABB3_1-30	30	14,854	8.34	4/3/2(2 inv)/1
DI2200005-1SUY (AB|C)	1588	*kaiA*	Q79V62_177-283	*kaiC*	Q79V60_485-518	34	2739	0.85	0/0/0/0
DI1200003-1QFN (A|B)	732	*grxA*	P68688_1-85	*nrdA*	P00452_732-761	30	7563	1.59	0/0/0/0
DI1200001-1R1R (A|D)	851	*nrdA*	P00452_335-729	*nrdB*	P69924_347-376	30	4425	0.78	0/1/0/0
DI1200006-5D0O (D|C)	2072	*bamD*	P0AC02_1-245	*bamC*	P0A903_30-85	56	726	0.38	0/0/0/0
DI1210006-4Z0U (B|E)	358	*rnhA*	A7ZHV1_1-155	*ssb*	P0AGE0_149-178	30	8735	0.31	0/0/0/0
DI1210004-3C94 (A|B)	320	*sbcB*	P04995_13-355	*ssb*	A0A0H3GL04_145-174	30	8735	0.11	0/0/0/0

^a^ In this column, bonds were assigned to the IDP EC residues based on PDBe PISA H-bond and salt bridge annotations. ^b^ The locations, bonds, and distances of invisible EC pairs could not be analyzed as they reside outside the sequence ranges with PDB coordinates. The numbers of such invisible EC pairs (inv) are indicated in brackets in the last column along with interface (IF) pairs, since they provide the explanation for the difference between the number of identified ECs and interface ECs.

## Supplementary Materials

Supplementary materials can be found at http://www.mdpi.com/1422-0067/19/11/3315/s1.
