# Supplementary material for: Co-Evolution of Intrinsically Disordered Proteins with Folded Partners Witnessed by Evolutionary Couplings
Source: Int J Mol Sci. 2018 Oct 25;19(11):3315. doi: 10.3390/ijms19113315 (PMC6274761; doi:10.3390/ijms19113315)
Supplement: Supplementary file 1 [file ijms-19-03315-s001.pdf]

Supplementary Table S1. Residue co-variation analysis of bacterial IDP-partner interactions from DIBS with the corresponding phylogenetic spread indicated.

**Complex**		**Folded partner**		**IDP**				**Gremlin analysis results**		**Features of the interaction**		
**DIBS ID - PDB ID (partner(s)|IDP chains)**	**IF area (Å²)**	**Gene name**	**Uniprot AC_region**	**Gene name**	**Uniprot AC_region**	**Length**	**# of seq. in PFAM 31 full**	**Coverage (seq/res)**	**ECs by Gremlin/ by EVcomplex/ Gremlin IDP ECs on IF/ in bonds [a]**	**Permanent/ Transient**	**Same gene neighborhood/ plasmid/operon?**	**Phylogenetic groups where interaction is present (ref)**
DI4200001 - 3B1K (BG|D)	1016.1	*gap2*	Q9R6W2_78-215	*cp12*	Q6BBK3_46-75	30	507	0.76	1/ 0/ 1/ 1	Transient	No	Most photosynthetic organisms [1]
DI2200001 - 3HPW (AB|C)	1483.2	*ccdB*	P62554_1-101	*ccdA*	P62552_37-72	36	395	4.06	7/ 0/ 6(1inv)/ 2	Transient	Yes	Free-living prokaryotes [2]
DI2200002 - 5CQX (AB|C)	1127.9	*mazF*	P0AE70_1-111	*mazE*	P0AE72_53-82	30	4349	3.00	9/ 1/ 5(4inv)/ 5	Transient	Yes	Free-living prokaryotes [2]
DI2200004 - 3M91 (AC|B)	1064.9	*mpa*	P9WQN5_46-96	*pup*	P9WHN5_21-64	44	511	2.19	5/ 0/ 2(2inv)/ 1	Transient	No	Not known
DI2200006 - 3M4W (AC|E)	2320.6	*rseB*	P0AFX9_220-318	*rseA*	P0AFX7_125-195	71	253	1.67	2/ 0/ 2/ 1	Transient	Yes	Gram negative bacteia [3]
DI1200004 - 1SC5 (A|B)	1640.9	*fliA*	O67268_1-236	*flgM*	O66683_1-88	88	1450	3.43	3/ 10/ 3/ 1	Transient	No	Flagellar bacteria
DI1210003 - 2A7U (B|A)	610.5	*atpH*	P0ABA5_1-134	*atpA*	P0ABB3_1-30	30	14854	8.34	4/ 3/ 2(2inv)/ 1	Permanent	Yes	All bacteria, mitochondria and chloroplasts
DI2200005 - 1SUY (AB|C)	1587.6	*kaiA*	Q79V62_177-283	*kaiC*	Q79V60_485-518	34	2739	0.85	0/ 0/ 0/ 0	Transient	Yes	Cyanobacteria [4]
DI1200001 - 1R1R (A|D)	851.4	*nrdA*	P00452_335-729	*nrdB*	P69924_347-376	30	4425	0.78	0/ 1/ 0/ 0	Permanent	Yes	Not known
DI1200003 - 1QFN (A|B)	731.9	*grxA*	P68688_1-85	*nrdA*	P00452_732-761	30	7563	1.59	0/ 0/ 0/ 0	Transient	Yes	Not known
DI1200006 - 5D0O (D|C)	2071.8	*bamD*	P0AC02_1-245	*bamC*	P0A903_30-85	56	726	0.38	0/ 0/ 0/ 0/	Permanent	No	Gram negative bacteria
DI1210006 - 4Z0U (A|D)	358.1	*rnhA*	A7ZHV1_1-155	*ssb*	P0AGE0_149-178	30	8735	0.31	0/ 0/ 0/ 0/	Transient	No	Widely present in Eubacteria
DI1210004 - 3C94 (A|B)	320.2	*sbcB*	P04995_13-355	*ssb*	A0A0H3GL04_145-174	30	8735	0.11	0/ 0/ 0/ 0/	Transient	No	Mainly Gammaproteobacteria
DI2210002 - 1YFN (AB|E)	843.1	*sspB*	P0AFZ3_1-118	*rseA*	P0AFX7_77-108	30	613	0.3	-/ 1/ 0/ 0/	Transient	No	Gram negative bacteria [3]
DI1200005 - 2W9R (A|B)	549.9	*clpS*	P0A8Q6_1-106	*dps*	P0ABT2_2-31	30	8622	0.33	-/ 0/ 0/ 0/	Transient	No	Not known
DI1200007 - 2N01 (B|A)	901.2	*virB9*	Q8PJB5_150-255	*virB7*	Q8PJB3_21-50	30	161	0.37	-/ 1/ 0/ 0/	Permanent	Yes	Gram-negative bacteria [5]
DI1200010 - 3Q8D (A|E)	278	*recO*	P0A7H3_80-242	*ssb*	P0AGE0_149-178	30	8735	0.26	-/ 0/ 0/ 0/	Transient	No	Widely present in Eubacteria
DI1200011 - 3UF7 (A|B)	404.6	*ung*	P12295_1-229	*ssb*	P0AGE0_149-178	30	8735	0.2	-/ 0/ 0/ 0/	Transient	No	Widely present in Eubacteria
DI1200013 - 4NL8 (A|C)	254.4	*priA*	A6TGC5_197-731	*ssb*	A0A0H3GL04_145-174	30	8735	0.06	-/ 2/ 0/ 0/	Transient	No	Widely present in Eubacteria
DI2200003 - 2FYM (AC|B)	919.3	*eno*	P0A6P9_2-432	*rne*	P21513_825-854	30	106	-	-	Transient	Yes	A subfamily of Gammaproteobacteria [6]
DI2200007 - 2XCB (AB|C)	518.6	*pcrH*	Q9I325_21-160	*pepD*	O50280_40-69	30	12	-	-	Transient	No	Several Gram-negative pathogenic bacteria
DI2200008 - 3GZ1 (AB|Q)	557.5	*ipgC*	P0A2U4_1-151	*ipaB*	P18011_47-76	30	20	-	-	Transient	Yes	Several Gram-negative pathogenic bacteria
DI2200009 - 1JYO (BD|E)	2503.9	*sicP*	P0CL16_2-116	*sptP*	P74873_35-139	105	8	-	-	Transient	Yes	Several Gram-negative pathogenic bacteria
DI2200010 - 3O6Q (AC|B)	2410.6	*spoIISA*	O34853_92-248	*spoIISB*	O34800_1-56	56	5	-	-	Transient	Yes	*Bacillus subtilis* strains
DI2200011 - 3KXY (AB|T)	2474.9	*exsC*	P26995_1-131	*exsE*	Q9I322_16-81	66	no PFAM	-	-	Transient	Yes	Several Gram-negative pathogenic bacteria
DI2200012 - 4JMF (BC|A)	2099.9	*spcS*	G3XD93_1-116	*exoT*	Q9I788_28-77	50	9	-	-	Transient	Yes	Several Gram-negative pathogenic bacteria
DI2210003 - 1L2W (AB|I)	2427.8	*sycE*	Q663P0_2-122	*yopE*	P08008_17-85	69	1	-	-	Transient	Yes	Several Gram-negative pathogenic bacteria
DI1200002 - 2IVZ (A|E)	689.3	*tolB*	P0A855_161-430	*col*	P09883_25-54	30	6	-	-	Transient	No	Escherichia coli strains
DI1200008 - 3GME (A|D)	837.6	*pnp*	A7ZS61_301-545	*rne*	A7ZKI9_1021-1061	41	106	-	-	Transient	Yes	γ-proteobacteria, α-proteobacteria and cyanobacteria
DI1200009 - 3O0E (B|M)	624	*ompF*	P02931_23-362	*col*	P09883_2-31	30	6	-	-	Transient	No	Gram negative bacteia
DI1200015 - 4AM9 (A|B)	586.9	*sycD*	O87496_21-163	*yopD*	Q9R2G2_45-74	30	12	-	-	Transient	Yes	Several Gram-negative pathogenic bacteria

DI1200016 - 4G6T (A|B)	1992.3	*shcA*	Q87UE6_1-125	*hopA1*	Q87UE5_21-102	82	no PFAM	-	-	Transient	Yes	Several Gram-negative pathogenic bacteria
DI1210001 - 3IAX (A|B)	574.3	*tolB*	P0A855_161-430	*caa*	P04480_1-107	107	17	-	-	Transient	No	*Escherichia coli* strains
DI1210002 - 3QDR (A|B)	632.2	*tolA*	P19934_302-421	*caa*	P04480_53-107	55	17	-	-	Transient	No	*Escherichia coli* strains
DI1210007 - 5CZF (D|A)	1423.8	*parE*	A0A0H3JHG3_2-92	*paaA2*	A0A0F6F6Q9_24-75	52	no PFAM	-	-	Transient	Yes	*Escherichia coli* strains
DI1210008 - 4GF3 (A|B)	937	*sycH*	Q7BTX0_1-141	*yopH*	P15273_21-63	43	3	-	-	Transient	Yes	Several Gram-negative pathogenic bacteria
DI1210009 - 1TTW (A|B)	655.3	*sycH*	Q7BTX0_1-138	*yscM2*	Q93KQ4_33-81	49	3	-	-	Transient	Yes	Several Gram-negative pathogenic bacteria

Color codes: Green background marks complexes with high-scoring ECs from Gremlin, yellow marks other complexes that could be analyzed with Gremlin. Orange background and "-" standing for high-scoring ECs by Gremlin indicates that the program stopped due to the alignments being insufficient for further analysis. Light grey background indicates the complexes that were not runned due to displaying <130 IDP homologs in PFAM 31 full alignments. [a] In this column bonds were assigned to the IDP EC residues based on PDBePISA H-bond and salt bridge annotations. The locations, bonds and distances of invisible EC pairs could not be analysed as they reside outside the sequence ranges with PDB coordinates. The numbers of such invisible EC pairs (inv) are indicated in brackets in the last column along with interface (IF) pairs, since they provide the explanation for the difference between the number of identified ECs and interface ECs.

Supplementary Table S2. PFAM entities for the IDP counterpart of analysed DIBS bacterial IDP-folded complexes.

DIBS ID - PDB ID (partner(s)|IDP chains)	IDP Gene name	Uniprot AC_region	Overlapping (O) or Neighboring (N) PFAM family?	PFAM name	PFAM identifier	# of seq. in PFAM 31 full
DI4200001 - 3B1K (BG|D)	*cp12*	Q6BBK3_46-75	O	CP12	PF02672	507
DI2200001 - 3HPW (AB|C)	*ccdA*	P62552_37-72	O	Ccda	PF07362	395
DI2200002 - 5CQX (AB|C)	*mazE*	P0AE72_53-82	O	MazE_antitoxin	PF04014	4349
DI2200004 - 3M91 (AC|B)	*pup*	P9WHN5_21-64	O	Pup	PF05639	511
DI2200006 - 3M4W (AC|E)	*rseA*	P0AFX7_125-195	O	RseA_C	PF03873	253
DI1200004 - 1SC5 (A|B)	*flgM*	O66683_1-88	O	FlgM	PF04316	1450
DI1210003 - 2A7U (B|A)	*atpA*	P0ABB3_1-30	N	ATP-synt_ab_N	PF02874	14854
DI2200005 - 1SUY (AB|C)	*kaiC*	Q79V60_485-518	N	ATPase	PF06745	2739
DI1200001 - 1R1R (A|D)	*nrdB*	P69924_347-376	N	Ribonuc_red_sm	PF00268	4425
DI1200003 - 1QFN (A|B)	*nrdA*	P00452_732-761	N	Ribonuc_red_lgC	PF02867	7563
DI1200006 - 5D0O (D|C)	*bamC*	P0A903_30-85	O	Lipoprotein_18	PF06804	726
DI1210006 - 4Z0U (A|D)	*ssb*	P0AGE0_149-178	N	SSB	PF00436	8735
DI1210004 - 3C94 (A|B)	*ssb*	A0A0H3GL04_145-174	N	SSB	PF00436	8735
DI2210002 - 1YFN (AB|E)	*rseA*	P0AFX7_77-108	O	RseA_N	PF03872	613
DI1200005 - 2W9R (A|B)	*dps*	P0ABT2_2-31	N	Ferritin	PF00210	8622
DI1200007 - 2N01 (B|A)	*virB7*	Q8PJB3_21-50	N	TcpQ	PF10671	161
DI1200010 - 3Q8D (A|E)	*ssb*	P0AGE0_149-178	N	SSB	PF00436	8735
DI1200011 - 3UF7 (A|B)	*ssb*	P0AGE0_149-178	N	SSB	PF00436	8735
DI1200013 - 4NL8 (A|C)	*ssb*	A0A0H3GL04_145-174	N	SSB	PF00436	8735
DI2200003 - 2FYM (AC|B)	*rne*	P21513_825-854	N	PNPase_C	PF12111	106
DI2200007 - 2XCB (AB|C)	*pepD*	O50280_40-69	O	YopD	PF05844	12

DI2200008 - 3GZ1 (AB|Q)	*ipaB*	P18011_47-76	N	T3SSipB	PF16535	20
DI2200009 - 1JYO (BD|E)	*sptP*	P74873_35-139	O	SicP-binding	PF09119	8
DI2200010 - 3O6Q (AC|B)	*spoIISB*	O34800_1-56	O	SpoIISB_antitox	PF14185	5
DI2200011 - 3KXY (AB|T)	*exsE*	Q9I322_16-81	no PFAM	no PFAM	no PFAM	no PFAM
DI2200012 - 4JMF (BC|A)	*exoT*	Q9I788_28-77	N	YopE	PF03545	9
DI2210003 - 1L2W (AB|I)	*yopE*	P08008_17-85	O	YopE_N	PF09020	1
DI1200002 - 2IVZ (A|E)	*col*	P09883_25-54	O	Cloacin	PF03515	6
DI1200008 - 3GME (A|D)	*rne*	A7ZKI9_1021-1061	O	PNPase_C	PF12111	106
DI1200009 - 3O0E (B|M)	*col*	P09883_2-31	O	Cloacin	PF03515	6
DI1200015 - 4AM9 (A|B)	*yopD*	Q9R2G2_45-74	O	YopD	PF05844	12
DI1200016 - 4G6T (A|B)	*hopA1*	Q87UE5_21-102	no PFAM	no PFAM	no PFAM	no PFAM
DI1210001 - 3IAX (A|B)	*caa*	P04480_1-107	N	Colicin	PF01024	17
DI1210002 - 3QDR (A|B)	*caa*	P04480_53-107	N	Colicin	PF01024	17
DI1210007 - 5CZF (D|A)	*paaA2*	A0A0F6F6Q9_24-75	no PFAM	no PFAM	no PAFM	no PFAM
DI1210008 - 4GF3 (A|B)	*yopH*	P15273_21-63	O	YopH_N	PF09013	3
DI1210009 - 1TTW (A|B)	*yscM2*	Q93KQ4_33-81	O	YopH_N	PF09013	3

Supplementary Table S3. High-scoring inter-protein ECs predicted by Gremlin

Protein pair	Contacting pair (IDP-partner) Gremlin numbering	Contacting pair (IDP-partner) PDB numbering	Gremlin Scaled Score | Probability	# of atomic contacts between EC pair; shortest distance (Å) based on PCA residue networks	Remarks	Distance (Å) calculated in PyMOL (which partner chain)
**3B1K** D | ABGH	29D-56Y	74D^{H}-133Y	1.93 | 0.91	no atomic contacts		13.078 (B)
CP12-GAPDH						
**3HPW** C | AB	28S-70S	64S^{H}-70S^{H}	1.95 | 1.00	no atomic contacts	A) 70S contacts 66A (i+2 in helix)	4.976 (A)
CcdA-CcdB	19V-67D	55V-67D^{H}	1.63 | 0.99	no atomic contacts	B) 55V contacts 64M (i-3)	6.74 (B)
	19V-66T	55V-66T	1.58 | 0.98	no atomic contacts	B) 55V contacts 64M (i-2)	4.507 (B)
	9K-26D	45K-26D	1.55 | 0.98	no atomic contacts	A) 45K contacts 24I (i-2);	5.797 (A)
	31D-75V	67D^{HS}-75V	1.44 | 0.97	no atomic contacts		7.496 (A)
	15G-47S	51G-47S	1.40 | 0.96	no atomic contacts	B) 51G contacts 46V (neighbour)	5.409 (B)
	1R-23D	N/A-23D	1.30 | 0.93	N/A		N/A
**5CQX** C | AB	10I-43T	N/A-43T^{H}	2.45 | 1.00	N/A		N/A
MazE-MazF	21W-79K	73W^{H}-79K	2.16 | 1.00	5; 3.726 (A)		-
	15L-43T	N/A-43T^{H}	2.01 | 1.00	N/A		N/A
	16H-61E	68H^{HS}-61E^{HS}	1.68 | 0.98	5; 2.566 (A)		-
	7V-38M	N/A-38M	1.56 | 0.97	N/A		N/A
	19I-77Q	71I^{H}-77Q^{H}	1.42 | 0.94	6; 2.831 (A)		-
	21W-48C	73W^{H}-48C	1.40 | 0.93	no atomic contacts		4.119 (A)
	14N-58Y	N/A-58Y	1.34 | 0.91	N/A		N/A
	21W-81I	73W^{H}-81I	1.30 | 0.89	10; 3.350 (A)		-
**3M91** B | AC	38Y-27K	N/A-72K^{HS}	2.45 | 1.00	N/A		N/A
Pup-Mpa	26V-35A	46V-80A	1.54 | 0.94	no atomic contacts	C) 46V contacts 77L (i-3 in helix)	9.817 (A)
	20L-36R	40L-81R^{HS}	1.48 | 0.92	3; 3.432 (C)		-
	44E-42L	N/A-87L	1.47 | 0.91	N/A		N/A
	12L-46V	32L^{H}-91V	1.45 | 0.91	no atomic contacts	C) 91V contacts 29R (i-3 in helix)	5.591 (C)
**3M4W** E | AC	59Q-7W	183Q^{H}-226W	1.91 | 0.98	no atomic contacts		17.571 (A)
RseA-RseB	46R-78V	170R-297V	1.67 | 0.94	no atomic contacts	297V contacts 176M and 177L	9.867 (A)
**1SC5** B | A	81K-175T	81K-175T	2.01 | 1.00	no atomic contacts	175T contacts 80V (neighbour)	6.601 (A)
FlgM-FliA	79V-194Q	79V^{H}-194Q^{H}	1.64 | 0.98	3; 3.421 (A)		-

	76D-183S	76D-183S	1.61 | 0.98	5; 2.772 (A)	-
**2A7U** A | B	19F-114A	19F-N/A	1.56 | 1.00	no X-Ray structure	N/A
AtpA-AtpH	26H-131R	N/A-N/A	1.46 | 0.99	no X-Ray structure	N/A
	13K-20E	13K[HS]-20E	1.44 | 0.99	no X-Ray structure	6.186 (B)
	15R-14A	15R-14A	1.30 | 0.98	no X-Ray structure	7.253 (B)

The ECs of each complex are indicated in the order of decreasing Gremlin scores. EC residues that fall into the extension regions and thus do not have PDB coordinates are marked by N/A within the 3rd column. For an EC with at least one such N/A residue atomic contacts and distances could not be obtained, so in the respective columns we also indicated N/A. EC residues with a chemical bond (H-bonds or salt bridges) noted in PISA are marked by H or S letters in superscript within the 3rd column. ECs with a Cα distance >10Å are considered as mistakenly predicted and are highlighted with grey background. The shortest distances of the ECs from the Protein Contacts Atlas (PCA) or in the absence of PCA contacts calculated by PyMOL are underlined with the respective partner chain indicated in brackets.

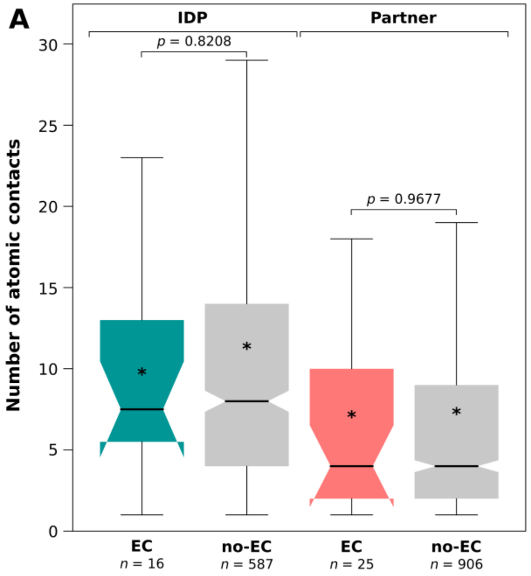

Supplementary Figure S1: Total number of atomic contacts compared between EC and no-EC residues. The total number of atomic contacts (with all contacting residues) has been calculated for all the residues with at least one such inter-chain atomic contact. The calculated totals have been compared between EC and non-EC residues for the IDPs and partners separately using Mann-Whitney U tests. Stars (*) indicated the average values of the datasets.

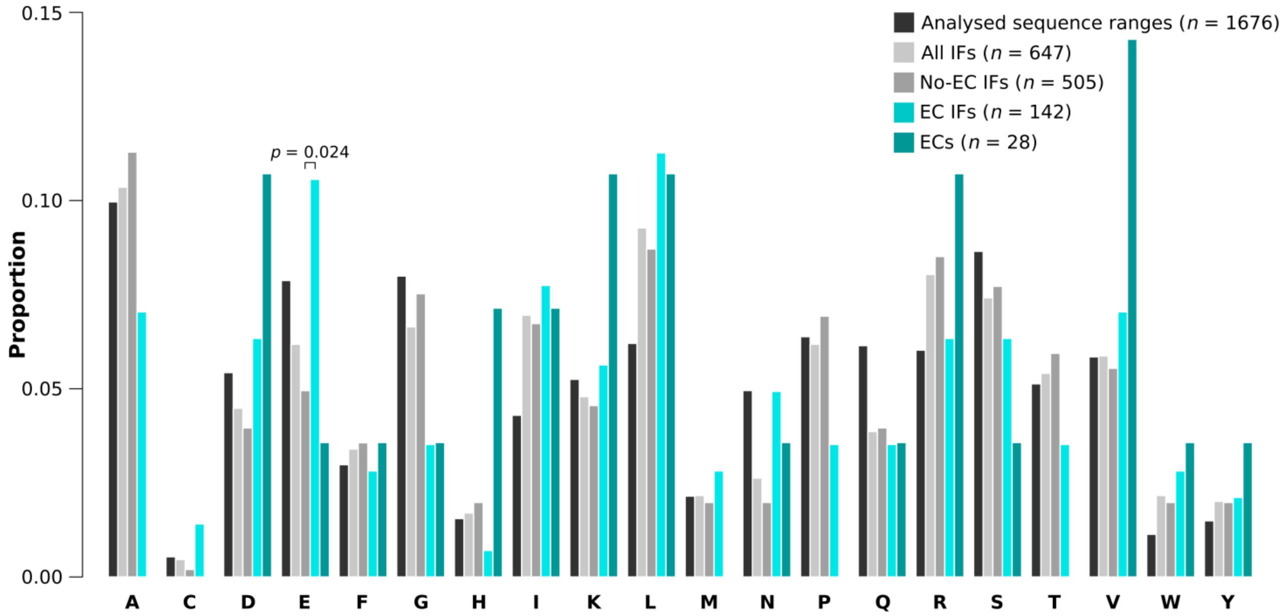

Supplementary Figure S2: The amino acid compositions of IDP EC-carrying interfaces and ECs. The amino acid compositions of trimmed protein segments (analysed sequence ranges), all interfaces (all IFs), interfaces with no ECs (no-EC IFs), EC-carrying interfaces (EC IFs) and EC residues of IDPs. Both EC-carrying interfaces and EC residues have been compared to all investigated interfaces. Also, EC interfaces were compared to no-EC interfaces. P-values are only indicated for amino acid proportion differences that were found significant in these comparisons by the built-in test of equal proportions of R.

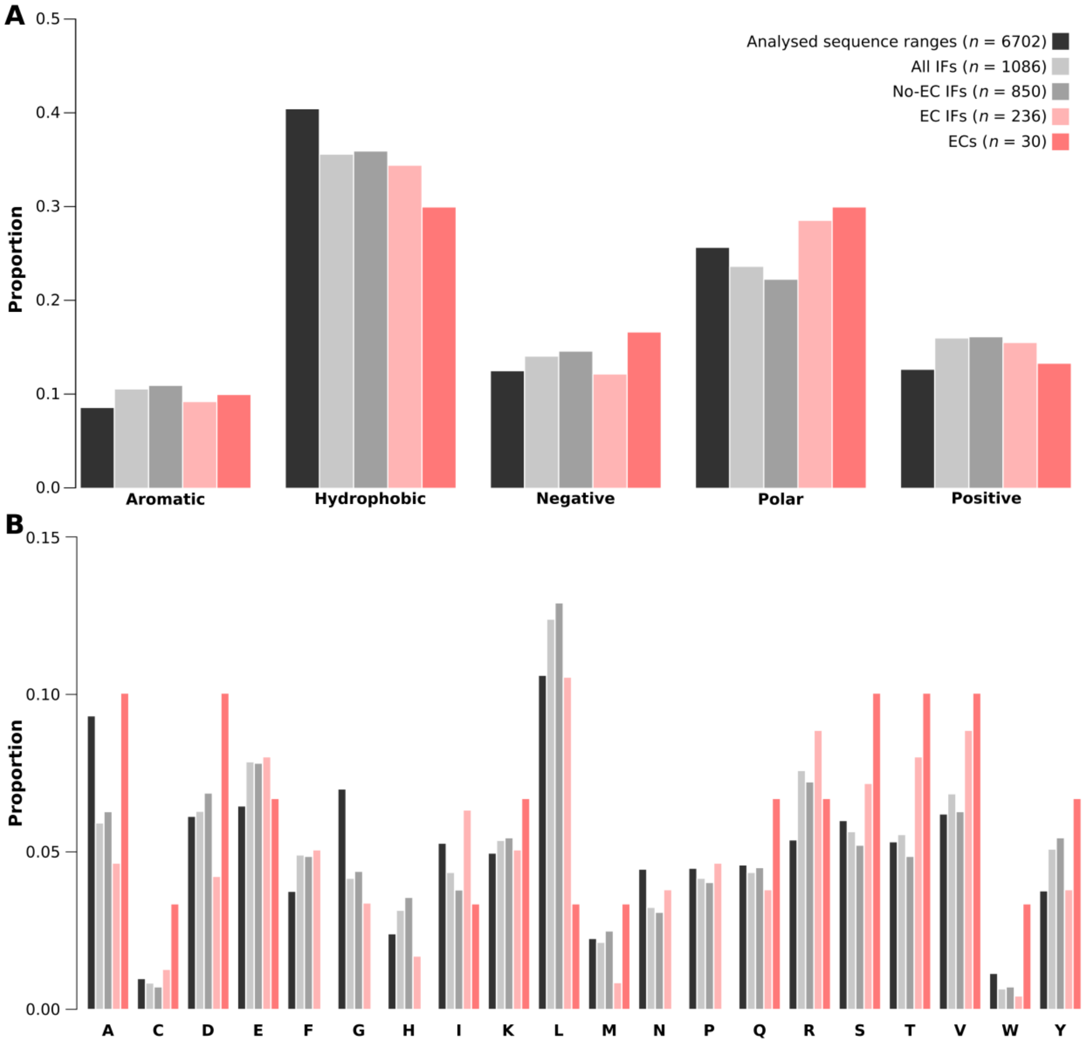

Supplementary Figure S3: The amino acid group and amino acid compositions of partner EC-carrying interfaces and ECs. The A) amino acid group and B) amino acid compositions of trimmed protein segments (analysed sequence ranges), all interfaces (all IFs), interfaces with no ECs (no-EC IFs), EC-carrying interfaces (EC IFs) and EC residues of partners. Both EC-carrying interfaces and EC residues have been compared to all investigated interfaces. Also, EC interfaces were compared to no-EC interfaces. Neither amino acid, nor amino acid group proportions have been found significantly different between the compared sets by the built-in test of equal proportions of R.

Supplementary references:

1. Lopez-Calcagno, P.E.; Howard, T.P.; Raines, C.A. The CP12 protein family: a thioredoxin-mediated metabolic switch? *Frontiers in plant science* **2014**, *5*, 9, doi:10.3389/fpls.2014.00009.
2. Pandey, D.P.; Gerdes, K. Toxin-antitoxin loci are highly abundant in free-living but lost from host-associated prokaryotes. *Nucleic acids research* **2005**, *33*, 966-976, doi:10.1093/nar/gki201.
3. Lima, S.; Guo, M.S.; Chaba, R.; Gross, C.A.; Sauer, R.T. Dual molecular signals mediate the bacterial response to outer-membrane stress. *Science* **2013**, *340*, 837-841, doi:10.1126/science.1235358.
4. Kondo, T.; Ishiura, M. The circadian clock of cyanobacteria. *BioEssays : news and reviews in molecular, cellular and developmental biology* **2000**, *22*, 10-15, doi:10.1002/(SICI)1521-1878(200001)22:1<10::AID-BIES4>3.0.CO;2-A.
5. Bayliss, R.; Harris, R.; Coutte, L.; Monier, A.; Fronzes, R.; Christie, P.J.; Driscoll, P.C.; Waksman, G. NMR structure of a complex between the VirB9/VirB7 interaction domains of the pKM101 type IV secretion system. *Proceedings of the National Academy of Sciences of the United States of America* **2007**, *104*, 1673-1678, doi:10.1073/pnas.0609535104.
6. Chandran, V.; Luisi, B.F. Recognition of enolase in the Escherichia coli RNA degradosome. *Journal of molecular biology* **2006**, *358*, 8-15, doi:10.1016/j.jmb.2006.02.012.
